# Diagnostic efficacy of cell block method for vitreoretinal lymphoma

**DOI:** 10.1186/s13000-016-0479-1

**Published:** 2016-03-17

**Authors:** Satoru Kase, Kenichi Namba, Daiju Iwata, Kazuomi Mizuuchi, Nobuyoshi Kitaichi, Yoshiaki Tagawa, Hiromi Okada-Kanno, Yoshihiro Matsuno, Susumu Ishida

**Affiliations:** Department of Ophthalmology, Hokkaido University Graduate School of Medicine, N15 W7, Kita-ku, Sapporo City, Hokkaido 060-8638 Japan; Department of Ophthalmology, Health Sciences University of Hokkaido, Sapporo, Japan; Department of Surgical Pathology, Hokkaido University Hospital, Sapporo, Japan

**Keywords:** Cytology, CD20, Cell block, Lymphoma, Uveitis, Masquerade syndrome

## Abstract

**Background:**

Vitreoretinal lymphoma (VRL) is a life- and sight-threatening disorder. The aim of this study was to analyze the usefulness of the cell block method for diagnosis of VRL.

**Methods:**

Sixteen eyes in 12 patients with VRL, and 4 eyes in 4 patients with idiopathic uveitis presenting with vitreous opacity were enrolled in this study. Both undiluted vitreous and diluted fluids were isolated during micro-incision vitrectomy. Cell block specimens were prepared in 19 eyes from diluted fluid containing shredding vitreous. These specimens were then submitted for HE staining as well as immunocytological analyses with antibodies against the B-cell marker CD20, the T-cell marker CD3, and cell proliferation marker Ki67. Conventional smear cytology was applied in 14 eyes with VRL using undiluted vitreous samples. The diagnosis of VRL was made based on the results of cytology, concentrations of interleukin (IL)-10 and IL-6 in undiluted vitreous, and immunoglobulin heavy chain gene rearrangement analysis.

**Results:**

Atypical lymphoid cells were identified in 14 out of 15 cell block specimens of VRL (positive rate: 93.3 %), but in 5 out of 14 eyes in conventional smear cytology (positive rate: 35.7 %). Atypical lymphoid cells showed immunoreactivity for CD20 and Ki67. Seven cell block specimens were smear cytology-negative and cell block-positive. The cell block method showed no atypical lymphoid cells in any patient with idiopathic uveitis.

**Conclusions:**

Cell block specimens using diluted vitreous fluid demonstrated a high diagnostic sensitivity and a low pseudo-positive rate for the cytological diagnosis of VRL. The cell block method contributed to clear differentiation between VRL and idiopathic uveitis with vitreous opacity.

**Electronic supplementary material:**

The online version of this article (doi:10.1186/s13000-016-0479-1) contains supplementary material, which is available to authorized users.

## Multilingual abstract

Please see Additional file 1 for translation of the Abstract into Japanese language.

## Background

Vitreoretinal lymphoma (VRL) is a life- and sight-threatening disorder. VRL is caused by primary VRL, known as primary intraocular lymphoma, and intraocular invasion from central nervous system lymphoma, as well as systemic lymphoma metastasizing to the retinochoroidal tissues. VRL is sometimes observed as a masquerade syndrome. It is indisputable that early diagnosis of VRL is mandatory to start adequate treatments and manage the patients from the early stage of the disease. VRL patients exclusively present with two ocular manifestations: vitreous cellular infiltration and subretinal tumor infiltration [1]. Actually, the opportunity to diagnose VRL seems to be increasing among uveitis patients according to recent multicenter analyses [2]. On the other hand, the clinical diagnosis of VRL is still not always feasible because there are no specific ophthalmological findings related to VRL. In the patients with severe vitreous opacity, laboratory tests using intraocular fluids are required to differentiate VRL from idiopathic uveitis particularly in patients presenting with vitreous opacity of unknown etiology. Historically, undiluted vitreous obtained by dry anterior vitrectomy should be optimal material, being exclusively used for the analysis of laboratory tests such as cytology, measurements of interleukins (IL), and further immunoglobulin heavy chain (IgH) gene rearrangement analysis. However, the amount of undiluted vitreous fluid that can be obtained safely is limited to a maximum of 1 mL.

Diffuse large B-cell lymphoma is histologically the most common subtype of lymphoma arising in the eye as VRL [3]. It was proposed that the detection of malignant lymphoma cells using cytological examination could have a definitive impact on making a diagnosis of VRL [4]. However, the recent study showed that the diagnostic power of conventional cytology was not high compared to other laboratory tests including the ratio of IL-10/IL-6 and IgH gene rearrangement analysis [2]. Therefore, the fact that the sensitivity of cytological examinations is low is the most serious problem. Recently, cytological examinations using other samples such as vitreous infusion fluids obtained in vitreous surgery may be useful to complement the differential diagnoses of VRL/uveitis. Matsuoka et al. reported that smear cytology using vitreous infusion fluid contributed to the diagnosis of VRL in 2 patients [5]. With recent advances in pathological techniques, cell block preparations using vitreous infusion fluids could also be used for the improvement of the diagnostic efficacy for VRL/uveitis [6, 7]. However, there have been no reports comparing the diagnostic utility for VRL between conventional smear cytology and cell block preparations.

In this study, the cytological diagnostic utility was compared between conventional smear cytology and cell block preparations in VRL patients. Moreover, cytological findings in cell block specimens were analyzed in VRL and idiopathic uveitis patients.

## Methods

This is a retrospective observational case study. Twenty eyes in 16 patients who had vitreous opacity with suspected VRL with or without subretinal infiltration were enrolled in this study (Table 1). They underwent micro-incision vitrectomy (23 or 25 gauge). Undiluted vitreous fluid was obtained during dry anterior vitrectomy without infusion, and then vitreous fluid diluted by balanced salt solution was obtained following the core vitrectomy. After the samples of undiluted vitreous were centrifuged, the concentrated cells were submitted for smear cytology, and the supernatant was assayed for IL concentrations. Diluted vitreous fluids were submitted for the analyses of cell block preparations and IgH gene rearrangement. IL concentrations were measured by enzyme-linked immunosorbent assay. The IgH gene rearrangement analysis was evaluated using the polymerase chain reaction (PCR) method. The diagnosis of VRL was made based on the results of ophthalmological findings as well as laboratory tests including cytological examination, IgH gene rearrangement analysis, and undiluted intravitreal concentrations of IL-10 and 6. In the differential diagnosis of VRL, the presence of atypical cells in the specimens was defined as positive in cytology. If monoclonal IgH gene rearrangement was detected, the result of IgH was evaluated as positive. High IL-10 concentrations (>100 pg/mL) and IL-10/IL-6 ratios (>1.0) were evaluated as positive based on a previous report [8]. Uveitis patients definitely diagnosed by clinicopathological examinations such as for Behcet’s disease, sarcoidosis, Vogt-Koyanagi-Harada disease, viral uveitis, acute retinal necrosis, infectious/post operative endophthalmitis, tuberculosis, and syphilis were excluded from this study.

**Table 1:** Clinical features and laboratory tests of vitreoretinal lymphoma and uveitis examined in this study

Case	Age (years)	Sex	Eye	Type	Smear cytology	Cell block cytology	IgH	IL-10 (pg/mL)	IL-6 (pg/mL)
1	51	M	R	PIOL	-	+	+	14,900	65
2	80	F	L	PIOL	-	+	+	308	108
3	71	M	R	PCNSL → IOL	-	ND	-	28	36.8
			L	PCNSL → IOL	+	+	-	268	11.8
4	79	F	R	Systemic → IOL	-	+	-	480	105
			L	Systemic → IOL	+	+	-	417	328
5	69	F	L	PIOL	+	+	-	21,100	56.5
6	82	M	R	PIOL	-	+	+	10	121
			L	PIOL	-	-	+	60	9
7	75	M	R	PIOL	+	+	+	90	60
			L	PIOL	-	+	+	60	9.3
8	74	F	L	PCNSL → IOL	+	+	+	1620	45.6
9	72	F	R	PCNSL → IOL	-	+	+	10	636
10	44	M	R	PCNSL → IOL	-	+	+	995	14.2
11	65	M	R	PCNSL → IOL	ND	+	-	25	13.6
12	48	F	L	PIOL	ND	+	+	128	25.2
13	79	F	R	Panuveitis	ND	-	NA	<10	263
14	82	F	L	Panuveitis	ND	-	NA	<10	513
15	77	M	L	Panuveitis	ND	-	NA	<10	47
16	59	F	R	Panuveitis	-	-	NA	<10	139

*PIOL* primary intraocular lymphoma, *PCNSL* primary central nervous system lymphoma, *IgH* monoclonality of immunoglobulin heavy chain gene rearrangement, *ND* not done, *NA* not applicable

### Cytological examinations

A total amount of about 1 mL of undiluted vitreous fluid collected at the time of surgery was immediately stored at 4°, which was sent fresh to a laboratory within about 1 h. After cytospin at 3000 rpm for 1 min, cellular pellets were automatically placed on the slides using auto-smear method (Sakura Finetek Japan Co.,Ltd). The slides were then fixed by 95 % ethanol for 30 min, and stained with a standard Papanicolaou procedure. According to previous reports [6, 7], cell block preparations were performed. Briefly, about 50 to 100 mL of diluted vitreous fluid was obtained, and was transferred to a laboratory at room temperature within 20 min after the isolation. The diluted vitreous fluids were centrifuged at 2500 rpm for 10 min. After centrifugation, the supernatants were carefully aspirated by pipette. Cellular pellets were mixed and fixed with 10 % paraformaldehyde overnight at room temperature. Formalin-fixed pellets following centrifugation at 3000 rpm for 3 min were then embedded in paraffin. There was not a further precipitation step with alcoholic fluids. Five-micrometer unstained sections were made and submitted for HE staining, and immunocytological examinations were conducted with antibodies against leukocyte common antigen (LCA) (Immynot), CD3 (DAKO), CD20 (Nichirei), and Ki67 (Mib-1, DAKO).

This study was approved by the Ethics Committee of Hokkaido University Hospital (015–0175).

### Statistical analysis

The Mann–Whitney *U* test was applied for the evaluation of significant differences in patients’ age and IL-6 concentrations between VRL and uveitis. A *p*-value of less than 0.05 was considered to show a significant difference.

## Results

### Clinical characteristics in IOL and idiopathic uveitis patients

**Fig. 1:**
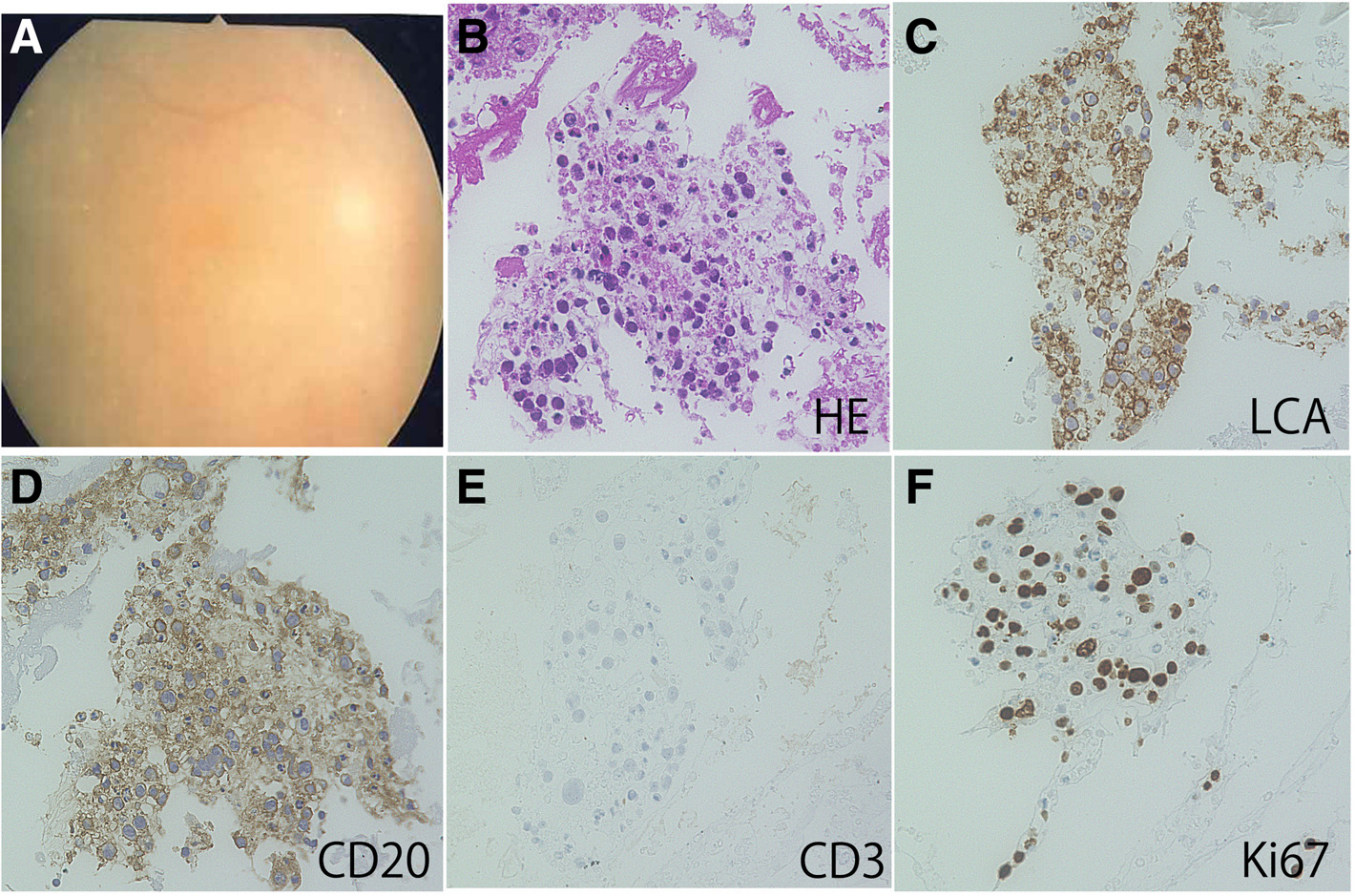
Fundus (**a**) and cell block (**b**-**f**) findings in a representative case of vitreoretinal lymphoma (Case 1, R). The fundus reveals dense diffuse vitreous opacity (**a**). HE staining demonstrates high cellularity of the collected cells with a high nuclear/cytoplasmic ratio and atypical nuclei, and hyper-chromatic cells (**b**). Immunocytochemically, the atypical lymphoid cells are positive for leukocyte common antigen (LCA) (**c**) and CD20 (**d**). Atypical lymphoid cells are negative for CD3 (**e**). Nuclear immunoreactivity for Ki67 is detected in the lymphoma cells (**f**)

**Fig. 2:**
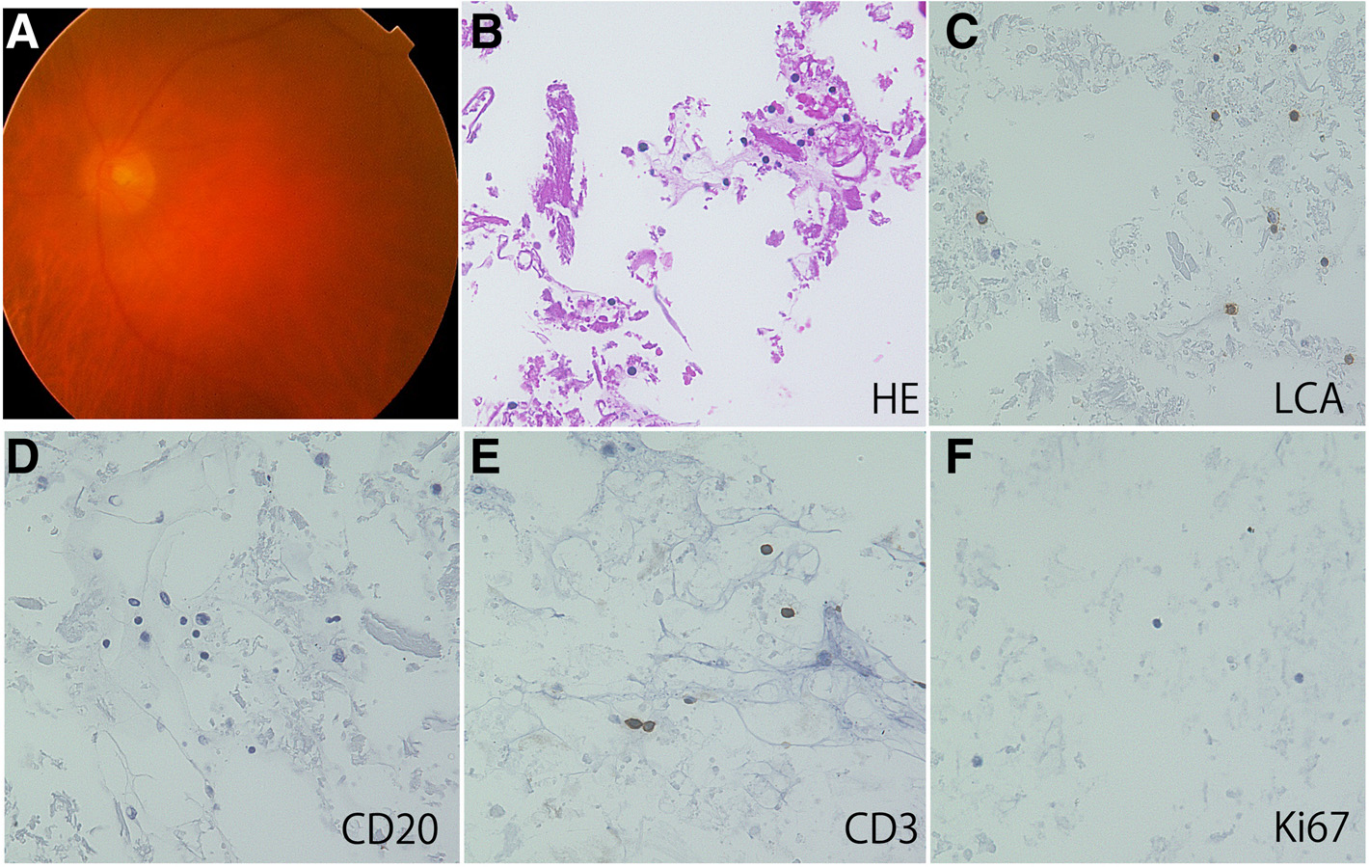
Fundus (**a**) and cell block findings (**b**-**f**) in a representative case of idiopathic uveitis (Case 16, R). The fundus reveals dense diffuse vitreous opacity (**a**). The cell block specimens present with low cellularity made up of small lymphocytes and macrophages in idiopathic uveitis. No necrotic background is noted in the specimens (**b**). Small lymphocytes are positive for leukocyte common antigen (LCA) (**c**). The lymphocytes are negative for CD 20 (**d**) and positive for CD3 (**e**). Nuclear immunoreactivity for Ki67 is not observed in the lymphocytes (**f**)

Table 1 summarizes clinical features and laboratory tests in VRL and uveitis patients examined in this study. Sixteen eyes in 12 VRL patients consisted of 6 males and 6 females. The patients’ ages ranged from 48 to 82 (mean: 67) years old. All patients showed vitreous opacity including 2 patients with subretinal infiltration before vitrectomy (Fig. 1a). Primary intraocular lymphoma occurred in 8 eyes in 6 patients. Five eyes in five patients were involved with intraocular invasion from primary central nervous system lymphoma. Two eyes in one patient showed systemic diffuse large B-cell lymphoma involving the inguinal region and femur metastasized to the eye. In idiopathic panuveitis, 4 patients presenting with blurred vision and diffuse vitreous opacity (Fig. 2a) underwent vitrectomy to differentiate from VRL. Four eyes in 4 uveitis patients consisted of 3 females and one male. The patients’ ages ranged from 59 to 79 (mean: 74) years old. There was no significant difference in patients’ ages between VRL and uveitis (*P* > 0.05). Among the 4 patients, clinical information of 2 patients (Case numbers 13 and 14 in Table 1) was partially reported in a recent publication [9].

### Cytopathological findings in smear cytology and cell block preparation

Findings of conventional smear cytology in VRL cases were increased cellularity, a large to medium cell size, marked nuclear irregularities, frequent apoptosis, lymphoglandular bodies, and necrosis [10]. The cell block specimens of VRL revealed abundant ghost cells, indicating a prominent necrotic background. The cellularity of the collected cells was high, many of which showed atypical lymphoid cells with hyper-chromatic nuclei and high nuclear/cytoplasmic ratio (Fig. 1b). Immunocytochemically, the atypical lymphoid cells were positive for LCA (Fig. 1c) and CD20 (Fig. 1d). In contrast, the atypical lymphoid cells were not positive for CD3 (Fig. 1e) whereas small CD3-positive lymphocytes were intermingled. Moreover, nuclear immunoreactivity for Ki67 was detected in a variety of lymphoma cells (Fig. 1f). Unlike the findings in VRL, the cell block specimens that showed low cellularity were made up of small lymphocytes and macrophages in idiopathic uveitis (Fig. 2b). No necrotic background was noted in the specimens. Small lymphocytes were positive for LCA (Fig. 2c) and CD3 (Fig. 2e), but not for CD20 (Fig. 2d). Nuclear immunoreactivity for Ki67 was not observed in the lymphocytes (Fig. 2f). Histological findings of cell block preparations on various ocular disorders will be presented in our future manuscript (Okada et al. manuscript in preparation).

### Diagnostic probability in laboratory tests

Since five eyes with VRL were positive for smear cytology, the positive rate in conventional smear cytology in the undiluted vitreous was 35.7 %. In contrast, lymphoma cells were detected in 14 out of 15 cell block specimens (positive rate: 93.3 %). Seven cell block specimens were smear cytology-negative, but cell block-positive. The IgH gene rearrangement analysis was positive in 10 eyes of 8 patients (positive rate: 67 %). Concentrations of IL-10 and IL-6 in VRL cases were 2531.2 ± 6158.7 and 102.8 ± 162.5 pg/mL, respectively. Nine eyes in 8 patients with VRL were IL-positive, indicating an IL-10/6 ratio of more than 1 and a high IL-10 concentration (positive rate: 56 %). In idiopathic uveitis, no eyes contained atypical lymphocytes in the cell block preparation (positive rate: 0 %). All idiopathic uveitis patients showed a high concentration of IL-6, measuring 47 to 513 pg/mL (mean: 240.5); however, in undiluted vitreous fluid, IL-10 concentration was undetectable (Table 1). Therefore, all idiopathic uveitis patients showed an IL-10/-6 ratio of less than 1 in the vitreous (positive rate: 0 %), while IL-6 concentrations in idiopathic uveitis were not significantly higher than VRL (*P* = 0.1). The IgH gene rearrangement analysis using the PCR method was not successfully conducted because of insufficient DNA isolation in idiopathic uveitis patients.

## Discussion

Cell block preparation was initially described by Bahrenburg using ascetic fluids at the end of the 19^th^ century [11]. The procedure of cell block preparation starts with the isolation and collection of cells from human body fluids, which are then submitted for fixation and further pathological analyses. Indeed, cell block preparations are applied for various fluids obtained from the human body, which has contributed to pathological diagnosis. Currently, vitreous fluids are known to be available in cell block preparations in the field of ophthalmology [7].

It has been proposed that cytological examination should be a gold standard to make a diagnosis of VRL [4]. However, recent studies proved that the positive rate in conventional smear cytology using the vitreous fluid is not high enough, ranging from 20 to less than 50 % [8, 12]. This means that the differential diagnoses between VRL/uveitis are still not easy, suggesting that some true VRL cases might be misdiagnosed as uveitis. Indeed, this study also demonstrated that, cytologically, malignant cells were detected in only 35.7 % of VRL patients based on conventional smear cytology, in which the positive rate determined in this study seems to be consistent with previous reports [8, 12].

This study also simultaneously compared the positive rate using cell block preparations with conventional smear cytology in VRL patients. Interestingly, more than 90 % of VRL patients were cell block-positive, indicating a high cytological diagnostic rate. Moreover, 7 eyes were negative for conventional smear cytology whereas cell block preparations detected malignant cells, indicating that cell block preparations could save patients who might not have been diagnosed with VRL using conventional cytological examinations.

In the present study, favorable diagnostic efficacy was achieved by using the cell block preparations from diluted vitreous fluids compared to conventional smear cytology from the undiluted vitreous fluid. There are some possible reasons considered as follows: 1) sampling site of cells: the number of viable lymphoma cells could be higher near the retina than in the anterior vitreous (usually obtained undiluted vitreous is the anterior vitreous), 2) sampling volume: micro-incision vitrectomy is safe and useful to isolate a sufficient volume of lymphoma cells to evaluate morphological features, 3) elapsed time before staining: it takes longer time to produce specimens from undiluted vitreous than diluted vitreous fluids because the former is initially collected and stored until finishing the subsequent core vitrectomy. This may promote morphological cell degeneration in the anterior vitreous, which causes diagnostic difficulty based on the cytology.

According to previous reports, a few authors have tried to examine cell block preparations in patients with VRL. Zaldivar et al. examined cell block preparations using vitreous fluids, and found that 6 preparations contained definite malignant cells from 14 VRL patients [7]. Intzedy et al. examined 7 patients with VRL, showing a positive cytology in 6 eyes using cell block preparations [6]. Raparia et al. analyzed cell blocks from 2 patients with VRL, in which one showed malignant cells in the specimen [13]. Therefore, not only the previous reports but also our study demonstrated favorable diagnostic rates using cell block preparations to make a diagnosis of VRL. In addition, this is the first report showing a favorable diagnostic rate using the cell block method compared to conventional smear cytology.

We have used the vitreous cutter with a small gauge needle such as 23 or 25 gauge to isolate human vitreous. Fine needle aspiration using such small needles can be available for cell block preparation in other malignancies including lung and mediastinal lesions [14]. However, since the vitreous histologically contains small number of cells with a lot of collagens as well as liquid, it is difficult to collect enough number of cells to submit cell block preparation by fine needle aspiration. Therefore, the process including not only cut of the collagen but also aspiration of fluid elements is mandatory to collect the sample in the vitreous, which have made cytological diagnosis difficult compared to other tissue malignancies.

This study also demonstrated that cell block specimens revealed characteristic malignant features containing a necrotic background as well as CD20-positive atypical B cells. In contrast, the background and presence of such atypical B cells could not be found in idiopathic uveitis, although small T-lymphocytes were noted in both VRL and uveitis. Therefore, the sensitivity and specificity of cell block preparations were 93.3 and 100 % for making a diagnosis of VRL, respectively. Moreover, Ki67-positive atypical lymphoid cells were clearly detected in cell block specimens of VRL, while no Ki67-positive cells were noted in uveitis specimens. These results suggest that not only cytoplasmic antigens but also nuclear antigens were well preserved in the cell block specimens following vitrectomy. Cell proliferation is high in VRL cells, indicating a more aggressive nature, which is consistent with a previous report [15]. Taken together, cell block preparations can be useful for differential diagnoses between VRL and uveitis based on morphological analyses as well as immunocytological examinations.

## Conclusions

Sixteen eyes in 12 patients with VRL, and 4 eyes in 4 patients with idiopathic uveitis presenting with vitreous opacity were examined by cell block preparations and conventional smear cytology. Cell block preparation using vitreous infusion fluid was more useful for diagnosis of vitreoretinal lymphoma than conventional smear cytology. Cell block methods also contribute to the differentiation of vitreoretinal lymphoma from idiopathic uveitis.

## Additional file

Additional file 1: Abstract in Japanese language. (DOCX 13.7 kb)

## Abbreviation

VRL: vitreoretinal lymphoma
