# Supplementary material for: Diagnostic efficacy of cell block method for vitreoretinal lymphoma
Source: Diagn Pathol. 2016 Mar 17;11:29. doi: 10.1186/s13000-016-0479-1 (PMC4797249; doi:10.1186/s13000-016-0479-1)
Supplement: Additional file 1: — Abstract in Japanese language. (DOCX 13.7 kb) [file 13000_2016_479_MOESM1_ESM.docx]

Additional non-English language abstract:

背景：眼内リンパ腫（VRL）は視力・生命予後に影響を及ぼす疾患である。本研究ではVRLの診断における細胞塊（セルブロック）標本の有用性を検討することを目的とした。

症例と方法：北海道大学病院眼科および他院を受診したVRL12例16眼、および硝子体混濁を呈した特発性ぶどう膜炎4例4眼を対象とした。小切開硝子体手術により、無希釈硝子体液および硝子体灌流液を採取した。19眼で、硝子体灌流液を用いたセルブロック標本を作製した。これらの標本を用いて特殊染色、およびB細胞マーカーである抗CD20抗体の免疫細胞化学的検討を行った。14眼では無希釈硝子体液を用いた塗抹細胞診を併せて行った。VRLの診断は、細胞診、無希釈硝子体液のIL-10, -6濃度、灌流液沈殿物から遺伝子再構成部位のモノクローナリティについて検討して行った。

結果：VRLの15眼中14眼でセルブロック標本にて悪性リンパ腫細胞が検出された（陽性率：93.3％）。一方、塗抹細胞診では5眼で悪性細胞が検出された（陽性率：35.7％）。塗抹細胞診で陰性、セルブロックで陽性を示したIOL症例は7例8眼であった。特発性ぶどう膜炎では、悪性細胞が検出された症例はなかった（陽性率：0％）。VRL細胞はCD20陽性を示したが、特発性ぶどう膜炎ではCD20陽性細胞は検出されなかった。

結語：硝子体灌流液を用いたセルブロック細胞診は、これまでの塗抹細胞診よりVRLの陽性率が高く、偽陽性も無かった。セルブロックは、VRLとぶどう膜炎との鑑別にも有用であることが示唆された。
